# Identification of Extended-Spectrum *β*-Lactamases* Escherichia coli* Strains Isolated from Market Garden Products and Irrigation Water in Benin

**DOI:** 10.1155/2015/286473

**Published:** 2015-12-07

**Authors:** Wassiyath Moussé, Haziz Sina, Farid Baba-Moussa, Pacôme A. Noumavo, Nadège A. Agbodjato, Adolphe Adjanohoun, Lamine Baba-Moussa

**Affiliations:** ^1^Laboratoire de Biologie et de Typage Moléculaire en Microbiologie, Faculté des Sciences et Techniques, Université d'Abomey-Calavi, 05 BP 1604 Cotonou, Benin; ^2^Laboratoire de Microbiologie et de Technologie Alimentaire, FAST, Université d'Abomey-Calavi, ISBA-Champ de foire, 01 BP 526 Cotonou, Benin; ^3^Centre de Recherches Agricoles Sud, Institut National des Recherches Agricoles du Bénin, Attogon, BP 884 Cotonou, Benin

## Abstract

The present study aimed at biochemical and molecular characterization of* Escherichia coli* strains isolated from horticultural products and irrigation water of Cotonou. The samples were collected from 12 market gardeners of 4 different sites. Rapid'* E. coli* medium was used for identification of* E. coli* strains and the antimicrobial susceptibility was performed by the agar disk diffusion method. The *β*-lactamases production was sought by the liquid acidimetric method. The genes coding for *β*-lactamases and toxins were identified by PCR method. The results revealed that about 34.95% of the analyzed samples were contaminated by* E. coli*. Cabbages were the most contaminated by* E. coli* (28.26%) in dry season. All isolated strains were resistant to amoxicillin. The penicillinase producing* E. coli* carried bla_TEM_ (67.50%), bla_SHV_ (10%), and bla_CTX-M_ (22.50%) genes. The study revealed that the resistance genes such as SLTI (35.71%), SLTII (35.71%), ETEC (7.15%), and VTEC (21.43%) were carried. Openly to the found results and considering the importance of horticultural products in Beninese food habits, it is important to put several strategies aiming at a sanitary security by surveillance and sensitization of all the actors on the risks of some practices.

## 1. Introduction

Fruits and leafy vegetables are interesting dietary source of nutrients, micronutrients, vitamins, and fiber for human beings, hence vital for health and fitness. In most of African towns, areas are often valorizing for market gardening. Indeed, due to the strong urbanization of many West African towns, the market gardening activity increases because it plays a vital role in the supply of populations with cheap and fresh products [1]. This activity also provides employment for many low-income families generally living under conditions of extreme poverty. Among the commonly produced vegetables, we can cite tomatoes, cabbage, lettuce, carrots, great nightshades, green pepper, basil, parsley, garden eggs, green beans, and leeks.

In developing countries such as Benin, gardeners use untreated waste water and manure as fertilizers for the production of their crops. This is a major contributing factor for the outbreak of diseases. In fact, the practices used by the market gardeners can foster a strong contamination of vegetables by microorganisms, some of which can be dangerous for the consumer. The differences in microbial profiles of various vegetables result largely from unrelated factors such as resident microflora in the soil, application of nonresident microflora via animal manures, sewage, or irrigation water, transportation, and handling by individual retailers [2]. Indeed, several cases of typhoid fever have been associated with eating contaminated vegetables grown in/or fertilized with contaminated soil or sewage [3].

Vegetables consumption is encouraged worldwide by governmental health agencies to protect against a range of illnesses such as cancers and cardiovascular diseases. However, leafy greens consumed raw are increasingly being identified as important cause of human pathogens that were traditionally associated with foods and/or animal origin [4]. Bacteria, viruses, and parasites on vegetables such as market garden products have been linked with illness [5]. But bacterial pathogens continue to be a major contributor to produce associated food-borne illnesses. Among those bacteria,* Salmonella *was the most commonly reported bacterial pathogen, accounting for nearly half of the outbreaks due to bacteria [6] followed by* E. coli*. Thus, it is established that enterobacteria account for more than half of the outbreaks due to bacteria. To explain this high level of contamination, the most frequently cited factors are water, soil manure, and animals [7].

Among the enterobacteria,* E. coli* is a common part of a normal microflora of man and most of the warm-blooded animals digestive tube. Nevertheless, some* E. coli* strains are reported to be a leading cause of extraintestinal (meningitis, urinary tract infections) and/or intestinal pathologies [8]. The pathogenicity of those strains can be due not only to their invasion proportion but also to toxins production. Enzymes displaying human cells cytopathic effect are part of such toxins [9]. To fight the pathogenic* E. coli* strains, many antibiotic molecules are often used worldwide. But, due to the high mutation capability and the worse use of such antibiotics, many cases of* E. coli* multiresistance strains are reported. Indeed, since the advent of antibiotics, many microorganisms such as* E. coli* have developed various strategies to thwart the potential of action of these molecules [10]. The most advised antibiotic against pathogenic* E. coli* strains is beta-lactams. The most advised antibiotic against pathogenic* E. coli* strains is beta-lactams (cephemes, monobactames, carbapenems, etc.) [11]. The resistance to beta-lactams is thus the main resistance mechanism among pathogenic* E. coli* strains. The phenomenon is reported to be increased because of broad spectrum antibiotics uses in medicine and in agriculture [12].

Unfortunately, in Cotonou (Benin) no data is available in regard to the microbiological quality of market garden product and the water used in its watering. So this study is aimed at filling this gap by the molecular characterization of extended-spectrum *β*-lactamases* E. coli* strains isolated from both market garden products and irrigation water collected in Cotonou.

## 2. Material and Methods

### 2.1. Samples Collections for Market Garden Products

**Figure 1:**
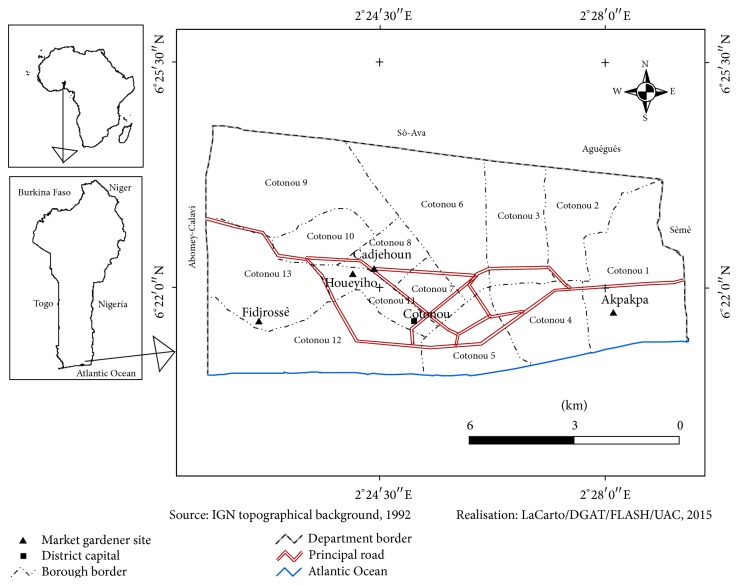
Overview of the garden sites.

Four kinds of market garden products (lettuce, cabbage, great nightshades, and carrots) were collected from 4 truck farming sites (Figure 1) (Fidjrossè Jacquot, Barrier Asecna, Akpakpa rifle-range, and ONIP Cadjèhoun) in Cotonou, Benin. The four sites were selected after a prior investigation. Per farming site, three gardeners were randomly selected among those who grow the four targeted products and two samples of each product were collected per gardener. The samples were collected both in dry season (January 2013-February 2013) and in rainy season (October 2013-November 2013). During the dry season, a total of 82 samples (22 lettuces, 22 cabbages, 22 great nightshades, and 16 carrots) were collected because the carrots samples that would be collected at ONIP Cadjèhoun were not found. During the rainy season, a total of 84 samples (22 lettuces, 16 cabbages, 22 great nightshades, and 22 carrots) were collected because the cabbages samples that would be collected at ONIP Cadjèhoun were missed. All the samples were collected in sterile Stomacher papers and then carried to laboratory in icebox at about 4°C.

### 2.2. Samples Collections for Irrigation Water

From the four sites listed above, three kinds of irrigation water (wells, ponds, and drilling) were collected for the irrigation. The water samples were only collected in dry season (January 2013-February 2013). Per farming site, three gardeners were randomly selected among those who grow the three targeted irrigation water samples and two samples of each water were collected per gardener. Then, a total of 22 irrigation water samples (8 wells, 6 ponds, and 8 drilling sites) were collected because the pond water samples that would be collected at Akpakpa rifle-range were missed. All the samples were collected in sterile Stomacher papers and then carried to laboratory in icebox at about 4°C.

### 2.3. Samples Microbiological Analysis

#### 2.3.1. Market Garden Products

Once carried at the laboratory, ten mL of each sample was aseptically poured into sterile bottles and diluted serially in distilled water up to a 10^−5^ dilution. Then, 1 mL of each tube dilutions 10^−4^ and 10^−5^ was mixed with 15 mL of Plate Count Agar (~45°C) and poured in sterile Petri dishes. After complete solidification, a second stratum (~4 mL) of the agar was added before being incubated at 30°C for 24 hours. The colonies that grew were counted per dish (30 to 300 colonies) and the values are expressed as colony forming units (CFU)/mL.

#### 2.3.2. Irrigation Water

Once carried at the laboratory, the microbial analysis was done using the filter membrane technique. After having filtered the water samples through a 0.45 *μ*m filter, the filter was deposited on Plate Count Agar in Petri dish and incubated at 37°C for 24 h. The colonies that grew were counted per dish (30 to 300 colonies) and the values are expressed as colony forming units (CFU)/mL using the formula cited above.

### 2.4. Characterization and Identification of* E. coli* Strains

#### 2.4.1. Market Garden Products

These strains were identified on Rapid'* E. coli*  medium according to BRD 07/01-07/93 standard. Briefly, 1 mL of suspension of each tube dilutions 10^−4^ and 10^−5^ has been paid aseptically in Petri dishes sterile. The melted and cooled (45°C) Rapid'* E. coli* medium was added to the inoculum (~15 mL per box). Then, the mixture was homogenized before being left for solidification and incubated at 44°C for 24 h. During the enumeration, the purple colonies with diameter ≤0.5 mm are characteristics of* E. coli* producing beta-D-glucuronidase and beta-D-galactosidase and blue colonies of diameter ≤0.5 mm are characteristics of* E. coli* producing only *β*-D-galactosidase. The number of seeds per gram of products analyzed is determined by calculation taking into account the dilution factor. The research of* E. coli* is completed by the indole production test [17].

#### 2.4.2. Irrigation Water

These germs are counted on the middle of triphenyl tetrazolium chloride (TTC) Tergitol 7 according to the standard BRD 07/01-07/93. A volume of 100 mL of water is filtered on the membrane filter; this last one is deposited on the middle of TTC that previously sank in Petri dishes after having been melted and cooled in a water bath at 45°C, at a rate of 15 mL per box. The boxes are then incubated in an oven at 44°C for 24 h to 48 h. The seeds of* E. coli* appear in colonies of diameter ≤0.5 mm and are yellowish with yellow halo. The alleged settlements are seeded on 15 mL of the medium EMB that sank in Petri dish after being melted and cooled in a water bath at 45°C. The boxes are then incubated at 44°C for 24 h. The purple colonies of diameter greater ≤0.5 mm are characteristics of* E. coli* on the agar EMB.

### 2.5. Antibiotics Susceptibility Testing* E. coli* Isolates

The antibiotics susceptibility pattern of the isolates was determined using the disk diffusion method on Mueller-Hinton agar (Oxoid, England). Inhibition zone diameter values were interpreted as recommended by the Antibiogram Committee of the French Society of Microbiology [11]. The 13 antibiotics (BioMérieux, France) used in this study are amoxicillin/clavulanic acid (20/10 *μ*g), cefotaxime (30 *μ*g), ceftriaxone (30 *μ*g), amoxicillin (30 *μ*g), imipenem (10 *μ*g), gentamicin (10 *μ*g), tobramycin (10 *μ*g), amikacin (30 *μ*g), kanamycin (30 *μ*g), nalidixic acid (30 *μ*g), ofloxacin (5 *μ*g), ciprofloxacin (5 *μ*g), and trimethoprim/sulfamethoxazole (1.25/23.75 *μ*g).

### 2.6. Phenotypic Detection of the Penicillinase

The production of penicillinase by the isolated* E. coli* strains was performed by tube acidimetric method [18]. Benzyl-penicillin (600 mg) was diluted in 400 *μ*L of distilled water before adding 300 *μ*L of aqueous phenol red solution (1%, w/v). The pH of the prepared benzylpenicillin solution was then adjusted to 8 with 1 M NaOH. The final 1 mL reaction volume was composed of suspension of two young isolated* E. coli* colonies and about 150 *μ*L of benzylpenicillin solution. The* E. coli *ATCC 25922 strains were used as a control. A yellow or orange colour within one hour at 37°C indicates penicillinase activity.

### 2.7. Phenotypic Detection of Extended-Spectrum Beta-Lactamase (ES*β*L)

The phenotypic detection of ESBL on the isolated* E. coli* strains was performed by double disk synergy test [19, 20]. In this test, the tested strains (10^6^ bacteria/mL) were flooded onto Mueller-Hinton according to the Antibiogram Committee of the French Society of Microbiology [11]. The test was performed with amoxicillin + clavulanic acid disc and the third-generation cephalosporins, namely, cefotaxime (30 *μ*g) and ceftriaxone (30 *μ*g). The amoxicillin + clavulanic acid disc was placed at the center of the inoculated Mueller-Hinton agar petri dish whereas the cefotaxime (30 *μ*g) and ceftriaxone (30 *μ*g) discs were placed at both sides (about 15 to 20 mm) of the amoxicillin + clavulanic acid disc. After 18 h incubation at 37°C, the enhancement of the zones of inhibition of any of the cephalosporin discs towards the clavulanic acid disc confirms the strains as an ESBL producer [21].

### 2.8. Detection of Resistance and Toxin Genes

Polymerase Chain Reactions (PCR) were performed on total DNA of all confirmed ESBL producer* E. coli *to detect genes encoding multidrug resistance (TEM, SHV, and CTX-M) and some virulence factors, namely, VT (verotoxin), that encode enterohemorrhagic* E. coli* (VTEC), LT (heat-labile enterotoxin) which encodes enterotoxigenic* E. coli* (ETEC), and SLTI (Shiga-like toxin I) and SLTII (Shiga-like toxin II) which encode Shiga toxin* E. coli* (STEC). The DNA template was extracted by suspending a loop of* E. coli *colony in 500 *μ*L sterile, pure water and boiling for 10 min at 95°C. The suspension was then centrifuged for 5 min at 12000 rpm, and 10 *μ*L of the supernatant was used as target DNA. DNA extracts were stored at −20°C until used.

The primers for bla_TEM_, bla_SHV_, and bla_CTX-M_ were used for multidrug resistance gene investigation by PCR amplification in 30 *μ*L containing for each 5 *μ*L of DNA, 0.5 *μ*M of each primer (F and R), 1.5 mM MgCl_2_, 250 *μ*M dNTPs, 1x PCR buffer (Invitrogen), and 1U Taq DNA polymerase (Invitrogen). The PCR program used for amplification consisted of the following: (i) bla_TEM_ (initial denaturation 94°C for 5 min followed by 30 cycles 94°C for 30 s, 52°C for 30 s, and 72°C for 1 min and a final elongation step 10 min at 72°C), (ii) bla_SHV_ (initial denaturation was performed at 96°C for 5 min, 30 cycles of 96°C for 15 s, 50°C for 15 s, and 72°C for 1 min and a final elongation step 10 min at 72°C), and (iii) bla_CTX-M_ (initial denaturation was performed at 95°C for 5 min, 35 cycles of 94°C for 1 min, 54°C for 1 min, and 72°C for 2 min and a final elongation step 10 min at 72°C). Four genes encoding virulence factors (VT, SLTI, SLTII, and LT) were searched in 25 mL containing 7.5 *μ*L of DNA, 12.5 *μ*L commercial 2x Master Mix Polymerase (BioLabs), and 0.5 *μ*L of each primer (F and R) at 0.2 *μ*mol/L. The PCR program used for amplification was 5 min at 95°C of initial denaturation, followed by 40 cycles of 45 s at 95°C, 45 s at 50°C, and 45 s at 72°C, and 10 min at 72°C for final extension. One control positive for Stx2 and LT was used. The primers sequences and the expected fragments are presented in Table 1.

PCR products (10 *μ*L) were visualized after electrophoresis at 150 V for 30 min on a 1.5% agarose gel containing ethidium bromide and visualized with UV transillumination. A 100 bp ladder standard was used as molecular weight ladder.

### 2.9. Data Analysis

The software Microsoft Office Excel 2010 was used for processing of the data. The software Epi Info 6 version 6.04 cfr January 1999 has helped to make the test of Chi-square. The test is considered statistically significant if *p* < 0.05.

## 3. Results

### 3.1. Environment of Market Gardening Sites

The observation of gardening areas environment displays that the same sites are cultivated by several market gardeners. Among those gardeners, some of them live inside the sites in huts with their family. Considering the market garden products, we note that once collected, they are sold to wholesaler but not directly to final consumers. After harvesting, the different garden products are primarily washed with pond or residual water observed neighborhood of the gardening areas.

### 3.2. Total Mesophilic Microbial Flora (FMT) and* E. coli* Count of Market Garden Products and Irrigation Water

The microbiological analysis focused on the total aerobic count and* E. coli* count from samples revealed a contamination rate variation accordingly (Table 2). Considering the market garden products, our data displays that the lettuce was the highest microbial load (2.6*∗*10^6^ CFU/g) followed by the carrots (2.5*∗*10^6^ CFU/g). For the irrigation water, we recorded that the water of wells was the most contaminated one (2.5*∗*10^6^) whereas the lowest colonies count was observed with the pool water (1.5*∗*10^6^ CFU/g). The same trends were observed in the enumeration of* E. coli* (Table 2). Thus, 0.65*∗*10^6^ CFU of* E. coli* were counted per gram of samples of lettuce. The water of pool was the least contaminated samples tested (0.062*∗*10^6^ CFU/g).

### 3.3. Identification of* E. coli* Strains

**Figure 2:**
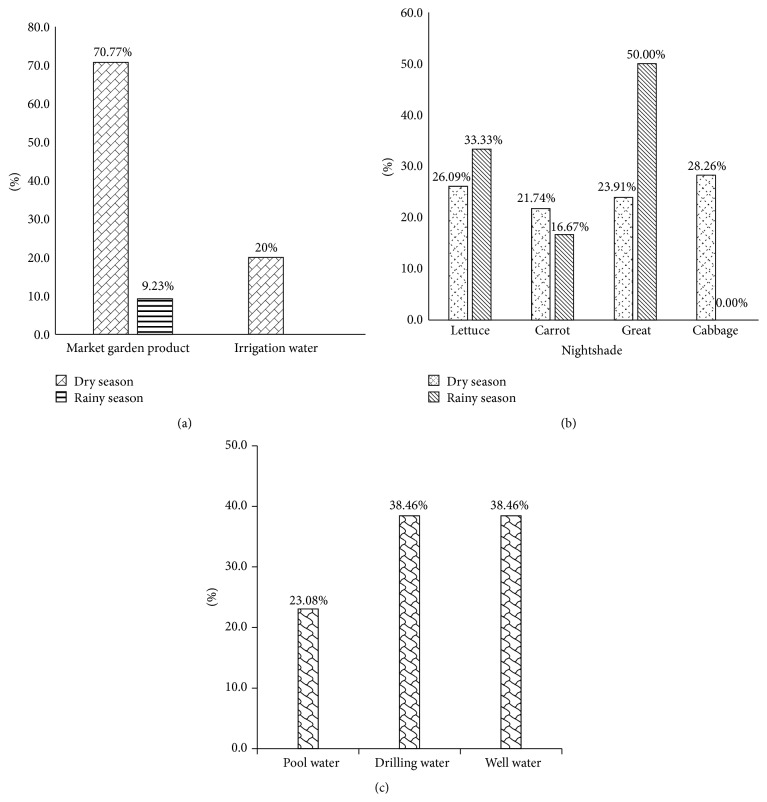
*E. coli* contamination rates of collected samples according to the season and the kind of samples. (a) Global contamination by* E. coli*. (b) Market garden products. (c) Irrigation water.

Among the 186 collected samples (market garden products and irrigation water), 65 were contained by* E. coli* strains. It appears that the market garden products were most contaminated compared to the irrigation water (Figure 2(a)). The market garden products global contamination patterns display high decreases from dry season (70.77%) to the rainy season (20%) (*p* < 0.00001).

Considering the market garden products, their* E. coli* contamination rate varies not only according to the kind of products (lettuce, carrots, cabbage, and great nightshades) but also according to the seasons (Figure 1). Indeed, our data shows that, among the 46* E. coli* strains isolated during the dry season, those of lettuce were the most contaminated ones (26.09%) followed successively by great nightshade (23.91%), cabbage (28.26%), and then carrots (21.74%) (Figure 2(b)). The difference was not statistically significant (*p* > 0.05). During the rainy season, six* E. coli* strains were isolated from the 84 collected samples. Three of the six (50%) strains were isolated from great nightshade samples, two (33.33%) were isolated from lettuce, and one (16.67%) was isolated from carrots.

For the irrigation water, out of the 22 collected samples, 13 of* E. coli* strains were isolated. Three (23.08%) strains were isolated from the pool water samples whereas 5 (38.46%) strains were both isolated from drilling and wells water (Figure 2(c)).

**Figure 3:**
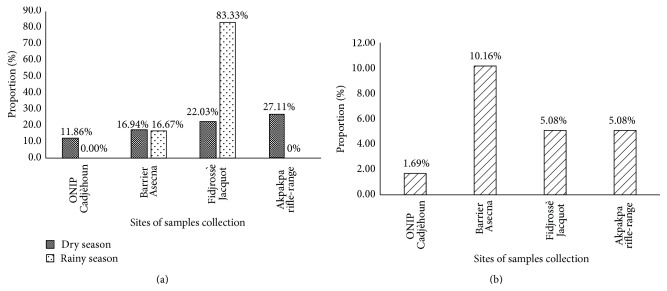
*E. coli* contamination rate of collected samples according to the site of samples collection. (a) Market garden products. (b) Irrigation water.

Focusing on the sampling site, we denote a variation of contamination rate from a collection site to another (Figure 3). Thus, it appears that market garden samples collected at Fidjrossè Jacquot during the rainy season were the most contaminated ones (*p* < 0.05). During the rainy season the sites of ONIP Cadjèhoun and Akpakpa rifle-range were free of* E. coli* contamination. But during the dry season, the entire collection site harbors the* E. coli* contamination at different level (Figure 2(a)). Considering the irrigation water, Figure 2(b) shows that those collected at Barrier Asecna were more contaminated (10.16%) than the ones collected in the three other sites (*p* < 0.05).

### 3.4. Susceptibility of Isolated* E. coli* Strains to Antibiotics

**Figure 4:**
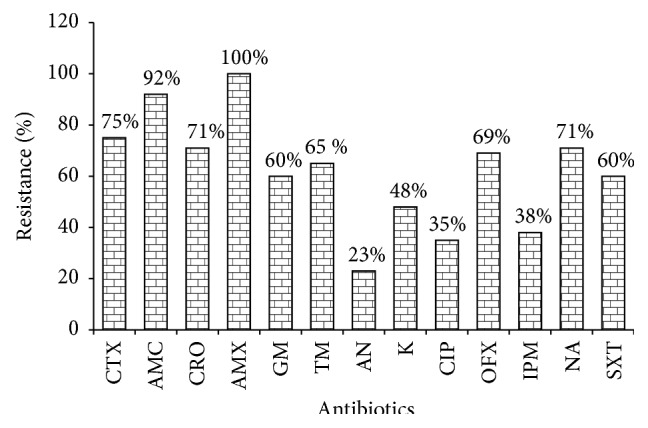
Resistance profile of the isolated* E. coli* strains to 13 antibiotics: amoxicillin (AMX), amoxicillin/clavulanic acid (AMC), cefotaxime (CTX), ceftriaxone (CRO), imipenem (IPM), gentamicin (GM), tobramycin (TM), amikacin (AN), kanamycin (K), nalidixic acid (NA), ofloxacin (OFX), ciprofloxacin (CIP), and trimethoprim/sulfamethoxazole (SXT).

The susceptibility of the 65* E. coli* strains isolated from our samples varies from an antibiotic to another (*p* < 0.05). Thus, it was observed that all strains were resistant to amoxicillin. Four of the tested antibiotics (amikacin, ciprofloxacin, imipenem, and kanamycin) display a resistance rate under 50% whereas the height remaining antibiotics (gentamicin, tobramycin, nalidixic acid, ofloxacin, trimethoprim/sulfamethoxazole, amoxicillin clavulanic acid, cefotaxime, and ceftriaxone) show their efficiency to less than 40% of the tested strains (Figure 4).

### 3.5. Phenotypic Detection of the Penicillinase and ESBLs

**Figure 5:**
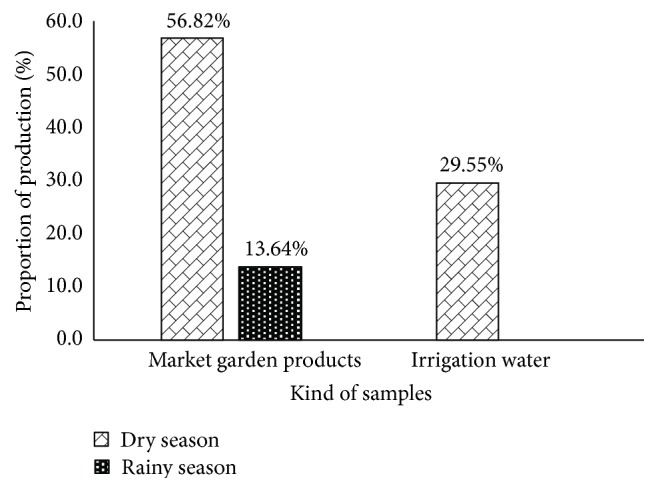
Phenotypical detection of penicillinase producer* E. coli* strains.

The phenotypical investigation shows that 67.69% of the 65 isolated* E. coli* strains produced penicillinase. The 44 strains producing penicillinase were composed of all strains of* E. coli* isolated from both market garden products and irrigation water (Figure 5). No strain of* E. coli* isolated was producing *β*-lactamase-producing plasmid to expanded-spectrum *β*-lactamases (also ESBLs).

### 3.6. Genomic Detection of the Penicillinase

The DNA of those strains was used to seek the presence of the bla_TEM_, bla_SHV_, and bla_CTX-M_ genes. The compilation of this investigation (Figure 5) displays that 67.50% of the tested strains carried the bla_TEM_ genes, 10% carried the bla_SHV_, and 22.50% carried the bla_CTX-M_ (Table 3). The distribution of the* E. coli* strains considerably varies according to the season (*p* < 0.0005).

### 3.7. Detection of Toxins Genes

The search of 4 toxins produced by pathogenic* E. coli* strains revealed that third of tested strains carried Shiga toxin 1 (35.71%) and Shiga toxin 2 (35.71%) while 7.15% carried LT gene and 21.43% carried VT gene (Table 4).

## 4. Discussion

The observation of the environment around the sites used for gardening revealed that several market gardeners live with their families on the sites. There was not latrine in all the investigated sites. Thus, to relieve themselves, they may have some unhealthy behaviors. Thus, to support this idea, Kifuani [22] found in a study led in Congo that the garden sites close to human houses are much more exposed to the microorganisms (viruses, protozoa, nematodes, etc.) contamination risks of market garden products and their irrigation water. Indeed, the microbiological quality focusing on the total mesophilic flora of the garden product and irrigation water collected in Cotonou is bad (Table 2). This high rate of contamination by bacteria may be explained by the fact that the farming practices adopted by the market gardeners favor permanent fecal contamination of irrigation water either directly (defecation) or indirectly through the streaming water. Such irrigation water is a reservoir of many cultures pathogenic microorganisms such as* E. coli*,* Salmonella*,* Vibrio cholerae*, and* Shigella *[23]. Also the risk of contamination increases by the fact that most of the gardeners use chicken droppings as soil fertilizer [24].

However, 34.95% of the collected samples were contaminated by* E. coli* strains. This proportion is lower than the 66% of* E. coli* strains isolated from human infections reported in the United States by Griffin [25]. Indeed, it was established that* E. coli* strains were more frequently isolated from vegetables and fruits than* Salmonella *[26]. The presence of Enterobacteriaceae may be due to poor hygiene conditions in which vegetables are usually grown [27]. Thus, the inappropriate consumption of vegetables can be a source of water diseases (cholera, bacillary dysentery, etc.). In dry season, the samples of market garden products are more contaminated (70.77%) by* E. coli* than those collected in the rainy season (9.23%) (*p* < 0.00001). These results suggest that the irrigation water may play a major role in the microbiological quality of vegetables [28]. Because, in the rainy season, garden produce was directly watered by rain, the rain may clean the market garden products and then increase the microbiological quality of leafy products. These results are similar to those obtained by Koffi-Nevry et al. [29] during their study on fecal contamination of irrigation water.

Among the samples of irrigation water, those collected from well (38.46%) and drilling (38.46%) were more contaminated than pool's ones (23.08%) (Figure 2(c)). This difference in proportion can be explained by the fact that the irrigation water is contaminated by sewage therefore subject to the presence of microorganisms [30]. In addition, the wells were the open wells and most well water is collected in tanks also in half-open tanks. This observation was also made in Abidjan by Koffi-Nevry et al. [29].

The* E. coli* strains were isolated from all the sampled market garden products (lettuce, carrot, cabbage, and great nightshade). These results are similar to those reported on several market garden products such as cabbage, carrots, lettuce, tomato, and pepper in Philippines [31]. The presence of* E. coli* in these samples confirms the close link between food-borne infections associated with the consumption of crudities [32]. Thus, plants and leafy variety are universally suspected as transmission way of food infections [3]. Among the garden products, our results display that carrot samples were weakly contaminated independently of the season. The low contamination rate of carrot samples can be explained by the fact that their edible parts are less exposed to direct contact of water during watering. However, this result seems contrary to that obtained by Vital et al. [31] when they found that the carrot was most contaminated product. In fact, Vital et al. [31] in their study used samples of fresh produce from open air markets and supermarkets. As vegetable leaves represent an appropriate surface of bacteria growth, our data shows that lettuce and great nightshade were highly contaminated by* E. coli* strains both in dry and in rainy seasons. Pathogenic bacteria are reported to survive on fresh vegetables for about 30 days [33]. We can then say that market garden leaves such as lettuce are the most contaminated produce of gardens as already reported in Côte d'Ivoire [29] and in Senegal [30].

Considering gardener sites, the market garden samples collected from Akpakpa rifle-range were more contaminated with* E. coli* in the dry season. Among the investigated sites, this site was unprotected and therefore is open to any type of movements including those of animals such as cattle for slaughtering in the same area. In addition, most of the housing near the site does not have latrines, so the site is currently used for defecation. Thus, the different manure (animal and human) may be the possible source of the high contamination level observed through streaming water [23]. In rainy season, the site closer to the beach, Fidjrossè Jacquot, was more contaminated whereas the site of ONIP was the less contaminated site independently of the season.

The results of antibiotic susceptibility of* E. coli* strains show that they were resistant to the majority of the 13 tested antibiotics at varying proportions. Thus, all the isolated* E. coli* strains were resistant to amoxicillin and 92% of those strains were resistant to the association of amoxicillin and clavulanic acid. These proportions are slightly higher than the 95.2% (amoxicillin) and 85.7% (amoxicillin and clavulanic acid) reported by Anago et al. [34] in Benin. These rates are higher than those obtained by some authors in developed countries; they observed resistance to amoxicillin oscillating between 42% [35] and 20.3% [36]. The high difference observed between developing countries and the case of Benin may be due to the fact that, in developing countries, the sale and administration of antibiotics are regulated. Thus, the proportion observed in general in our study for this antibiotic would find its explanation in a misuse of the antibiotic often sold on the street without a medical prescription [37]. This situation is observed not only in Benin but in most of the developing countries such as Senegal [38]. But we should also note that selection pressure is exerted in both the medical field and agricultural field [39]. For the other antibiotics such as ceftriaxone, higher resistance strains rates (71%) compared to those recorded in several studies conducted elsewhere in Africa were observed [34, 40]. The strong resistance proportion of the tested germs to antibiotics observed in this study also confirms the increase of resistance in* E. coli*.

It was noticed that the majority of* E. coli* (67.69%) strains produce penicillinase. Among those strains, 67.50%, 10%, and 22.50% harbor, respectively, bla_TEM_, bla_SHV_, and bla_CTX-M_ genes. The presence of genes bla_TEM_, bla_SHV_, and bla_CTX-M_ confirms the resistance of* E. coli* to the *β*-lactam antibiotics in this study. Indeed, the resistance to *β*-lactam antibiotics significantly increases over the two decades and the presence of bla_TEM_ gene is due to the resistance to third-generation antibiotics that cause secretion of *β*-lactamases [41]. None of the isolated strains produce expanded-spectrum *β*-lactamases (ESBLs). There were few strains isolated from food sector that produce ESBLs [42]. But in clinical area high rates of resistance were reported all over the world [34]. Originally, the group CTX-M conferred for Enterobacteriaceae a highest level of resistance for cefotaxime, cefepime, aztreonam, and ceftazidime [43]. In specialty hospital at India [44], 14% of bla_SHV_ and 50% of bla_CTX-M_ were found by* E. coli* ESBLs. From University Hospital in Turkey, bla_CTX-M_ was carried by 60.97% of* E. coli* ESBLs [45]. These rates were higher than the rate obtained in this study. Thus, origin of sample collection may play an important role in the resistance profile of given strains. Indeed, clinical isolated strains are more in contact with the antibiotics than the food isolated.

The enterohemorrhagic* Escherichia coli* (EHEC: STEC and VTEC) are responsible for various infections ranging from watery diarrhea to hemorrhagic colitis, which can progress to hemolytic uremic syndrome in young children or thrombotic microangiopathy in adults [46]. Moreover, strains STEC and ETEC are responsible for severe diarrhea in humans and animals [47]. In this study, the isolated* E. coli* strains were secreting Shiga toxin at levels of 35.71% (Stx1) and 35.71% (Stx2) (Table 4). In the dry season, STEC were found to be Stx2 of 21.43% and 21.43% of Stx1. Stx2 is more virulent than Stx1 [49]. In a study by Nataro et al. [47] on clinical strains, 0% and 0.2% of* E. coli* strains were, respectively, STEC and ETEC. Another study in Brazil on samples of infant diarrhea observed rates (1.2%) ETEC and (0.7%) STEC [40]. These types of strains are related to the production of cholera toxins [48]. One study made in Brazil by Oliveira et al. [49] showed that the prevalence of STEC ranged from 16% to 51.5%, 0 to 50%, and 46.7% to 73.3%, respectively, for beef cattle, dairy cattle, and goats, depending on the farm. Oliveira et al. [49] confirmed that the broad range of isolation of STEC observed in our study is probably associated with some farm management practices such as the administration of antibiotics, inappropriate manipulations of diet, and use of noncontaminated food and water. A rate of 7.15% of isolated* E. coli* strains was secreting heat-labile enterotoxin therefore (ETEC). ETEC, in contrast, is pathogenic across all age groups, but it is most common among infants in developing countries, because immunity is acquired from repeated exposure [47]. Irino et al. [50] who conducted a study on dairy cattle in Brazil assert that the presence of STEC (56.4% for Stx1 and 40.6% for Stx2) may be due to food contamination and failure water during handling by the farmer methods. These different rates observed may be due to the diversity of the origin of strains and geographic variation [47]. A rate of 8.33% of the strains was both STEC/VTEC. This unusual combination of virulence factors gives these strains a large pathogenicity [51]. These various results obtained in this study show that the consumption of garden produce raw or little cooked is very dangerous to humans.

## 5. Conclusion

The food poisonings are a subject of growing concern in the field of public health. This study was designed to bring new knowledge to the biochemical and molecular characteristics of* E. coli* strains isolated from irrigation water and market garden products sold in Cotonou, Benin.

This study has allowed us to determine the biochemical and molecular characters of* E. coli* strains. Thus, the microbiological quality of market garden products collected during the rainy season is better than those collected during the dry season. The irrigation water plays an important role in the contamination of market garden products. Most of the strains isolated produce penicillinase. An annual inspection committee is appropriate for the quality microbiology of the market garden products and water of watering on the sites.

## Figures and Tables

**Table 1:** Primers used to search genes in this study.

	Target genes	Primers	Primers sequences (5′ → 3′)	Amplicon size (bp)	Reference
*E*. *coli* multirestant	bla_TEM_	OT-F OT-R	5′-TTGGGTGCACGAGTGGGTTA-3′ 5′-TAATTGTTGCCGGGAAGCTA-3′	467	[13]

*E*. *coli* multirestant	bla_SHV_	SHV-F SHV-R	5′-CGCCGGGTTATTCTTATTTGTCGC-3′ 5′-TCTTTCCGATGCCGCCGCCAGTCA-3′	1017	[14]

*E*. *coli* multirestant	bla_CTX-M_	CTX-F CTX-R	5′-CGCTTTGCGATGTGCAG-3′ 5′-ACCGCGATATCGTTGGT-3′	550	[13]

EHEC	VT	VT-F VT-R	5′-GAGCGAAATAATTTATATGTG-3′ 5′-TGATGATGGCAATTCAGTAT-3′	518	[15]

STEC	SLTI (Stx1)	SLTI-F SLTI-R	5′-GAAGAGTCCGTGGGATTACG-3′ 5′-AGCGATGCAGCTATTAATAA-3′	150	[16]

STEC	SLTII (Stx2)	SLTII-F SLTII-R	5′-TTAACCACACCCACGGCAGT-3′ 5′-GCTCTGCATGCATCTCTGGT-3′	255	[16]

ETEC	LT	LT-F LT-R	5′-GCGACAAATTATACCGTGCT-3′ 5′-CCGAATTCTGTTATATATGT-3′	315	[16]

VT: verotoxin, EHEC: enterohemorrhagic *E*. *coli*, LT: heat-labile enterotoxin, ETEC: enterotoxigenic *E*. *coli*, SLTI: Shiga-like toxin I, SLTII: Shiga-like toxin II, and STEC: Shiga toxin *E*. *coli*.

**Table 2:** Total mesophilic count and *E*. *coli* count of market garden products and irrigation water collected in Cotonou.

Samples	FMT (CFU/g)	*E*. *coli* (CFU/g)
Horticultural products		
Lettuce	2.6 *∗* 10^6^	0.65 *∗* 10^6^
Carrot	2.5 *∗* 10^6^	0.44 *∗* 10^6^
Great nightshade	2.3 *∗* 10^6^	0.52 *∗* 10^6^
Cabbage	2.4 *∗* 10^6^	0.54 *∗* 10^6^
Water of watering		
Pool	1.5 *∗* 10^6^	0.062 *∗* 10^6^
Well	2.5 *∗* 10^6^	0.83 *∗* 10^6^
Drilling	1.8 *∗* 10^6^	0.66 *∗* 10^6^

**Table 3:** Distribution of the penicillinase genes carried by strains of *E*. *coli* according to the season.

	Dry season		Rainy season	Total
	Market garden products	Water of watering	Market garden products	
bla_TEM_	42.50%	20%	5%	67.50%
bla_SHV_	10%	0%	0%	10%
bla_CTX-M_	20%	0%	2.50%	22.50%

Total	72.50%	20%	7.50%	100%

**Table 4:** Distribution of the toxins genes carried by strains of *E*. *coli* according to the season.

	Dry season		Rainy season	Total
	Market garden products	Water of watering	Market garden products	
STEC (Stx1)	21.43%	0%	14.28%	35.71%
STEC (Stx2)	21.43%	0%	14.28%	35.71%
ETEC	7.15%	0%	0%	7.15%
VTEC	0%	14.28%	7.15%	21.43%
Total	50%	14.28%	35.71%	100%

VT: verotoxin, VTEC: enterohemorrhagic *E*. *coli,* LT: heat-labile enterotoxin, ETEC: enterotoxigenic *E*. *coli,* Stx1: Shiga-like toxin I, Stx2: Shiga-like toxin II, and STEC: Shiga toxin *E*. *coli*.
